# Three-year risk prediction of aortic stenosis using routine medical records: derivation and validation in 919 954 individuals from two cohorts

**DOI:** 10.1093/ehjdh/ztag035

**Published:** 2026-03-02

**Authors:** Ben O Petrazzini, Waqas A Malick, Stamatios Lerakis, Lori B Croft, Ghislain Rocheleau, Robert S Rosenson, Ron Do

**Affiliations:** The Charles Bronfman Institute for Personalized Medicine, Icahn School of Medicine at Mount Sinai, New York, NY, USA; Department of Genetics and Genomic Sciences, Icahn School of Medicine at Mount Sinai, New York, NY, USA; The Windreich Department of Artificial Intelligence and Human Health, Icahn School of Medicine at Mount Sinai, New York, NY, USA; Deep Medicine, Nuffield Department of Women’s and Reproductive Health, University of Oxford, Oxford, UK; Metabolism and Lipids Program, Mount Sinai Fuster Heart Hospital, Icahn School of Medicine at Mount Sinai, New York, NY, USA; Mount Sinai Fuster Heart Hospital, Icahn School of Medicine at Mount Sinai, New York, NY, USA; Department of Cardiology, Mount Sinai Morningside Hospital, Icahn School of Medicine at Mount Sinai, New York, NY, USA; Mount Sinai Fuster Heart Hospital, Icahn School of Medicine at Mount Sinai, New York, NY, USA; The Charles Bronfman Institute for Personalized Medicine, Icahn School of Medicine at Mount Sinai, New York, NY, USA; Department of Genetics and Genomic Sciences, Icahn School of Medicine at Mount Sinai, New York, NY, USA; The Windreich Department of Artificial Intelligence and Human Health, Icahn School of Medicine at Mount Sinai, New York, NY, USA; Metabolism and Lipids Program, Mount Sinai Fuster Heart Hospital, Icahn School of Medicine at Mount Sinai, New York, NY, USA; The Charles Bronfman Institute for Personalized Medicine, Icahn School of Medicine at Mount Sinai, New York, NY, USA; Department of Genetics and Genomic Sciences, Icahn School of Medicine at Mount Sinai, New York, NY, USA; The Windreich Department of Artificial Intelligence and Human Health, Icahn School of Medicine at Mount Sinai, New York, NY, USA

**Keywords:** Risk prediction, Aortic stenosis, Primary prevention

## Abstract

**Aims:**

All-cause mortality ranges between 33% and 42% for individuals with untreated moderate to severe aortic stenosis (AS). Transcatheter aortic valve replacement makes this a treatable condition, if identified early. Machine learning-based tools show great promise to predict cardiovascular outcomes.

**Methods and results:**

We developed and validated a machine learning model for 3-year prediction of AS risk (ASrisk) using serum biomarkers and vital sign measurements. We then evaluated the tool’s capacity to identify diagnoses of AS sequelae, echocardiographic outcomes in individuals not diagnosed with AS, as well as enrichment and 3-year aortic valve area reduction in individuals with high ASrisk. Among 919 954 participants, 429 996 were from the Mount Sinai Data Warehouse (MSDW) [2179 (0.5%) AS cases] and 489 958 were from the UK Biobank [5066 (1%) AS cases]. Odds ratio (OR) of AS sequelae increased quantitatively with ascending deciles of ASrisk [OR 1.63 (95% CI 1.60–1.67) in MSDW]. Increasing ASrisk by 1 SD resulted in higher odds of echocardiographic findings in undiagnosed individuals [OR 1.88 (95% CI 1.71–2.06) for Doppler velocity index, OR 2.50 (95% CI 2.36–2.64) for aortic valve area, and OR 2.61 (95% CI 1.89–2.71) for mean gradient]. Three years after risk assessment, individuals with ASrisk > 0.95 show an 11-fold enrichment for AS diagnosis in both cohorts and an average reduction in aortic valve area of 0.42 cm^2^.

**Conclusion:**

ASrisk can predict risk of AS 3 years ahead of diagnosis in the general population.

## Graphical Abstract

**Graphical Abstract:**
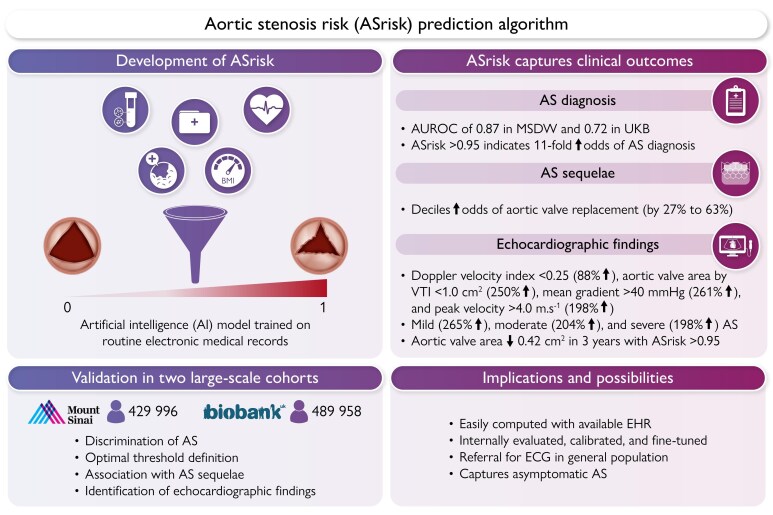

## Introduction

Degenerative calcific aortic valve stenosis (AS) is a condition associated with ageing and is increasing in prevalence globally as lifespans prolong worldwide.^1^ As AS severity progresses from mild to severe, morbidity and mortality increase as well, with a significant escalation seen once a patient has been diagnosed with moderate AS.^2^ Four-year mortality rates for patients with untreated AS ranged from 33% to 42% for moderate to severe AS, respectively. With the advent of transcatheter aortic valve replacement (AVR), AS is a treatable condition and can reduce mortality in patients with severe AS.^3–5^ However, despite these technological advancements, the rates of AVR in patients with AS remains underutilized.^6^

Detection of AS can enable the initiation of potential preventive measures such as controlling cardiovascular risk factors and early interventions prior to the patient developing significant risk from comorbidities. Although AS has not been traditionally thought of as a preventable condition, both hypertension and hypercholesterolaemia have been associated with increased risk of development and progression of AS.^7,8^ Additionally, elevated lipoprotein(a) [Lp(a)] has been found to have a strong association with AS,^9^ and a clinical trial is underway to determine whether Lp(a) lowering can slow progression of AS (NCT05646381). Therefore, early risk assessment and diagnosis for AS may be clinically valuable for instituting preventive therapies and allowing for earlier intervention prior to patients becoming high-risk for AVR.

Machine learning models have been developed to accurately predict coronary artery disease (CAD) using electronic health record (EHR) data,^10^ and also to quantify the amount of CAD burden using in-silico scores.^11^ Similarly, machine learning models trained on electrocardiograms and echocardiograms have shown predictive capacity to detect and quantify AS.^12–18^ Serum biomarkers and vital sign measurements, routinely collected in digitalized health systems, can possibly capture clinical factors of AS risk. Machine learning models trained on routinely collected data can provide a scalable tool for large-scale assessment of AS risk. Such an in-silico score for AS can help identify patients at risk for and quantify the severity of AS prior to any diagnostic testing. In this study, we used serum biomarkers and vital sign measurements to develop and validate an in-silico score to predict the 3-year risk of AS and quantify severity of AS. We evaluated the association of the in-silico score with echocardiographic data and AS sequelae. Finally, we estimated the enrichment of AS diagnoses and 3-year reduction of aortic valve area in individuals with high ASrisk (>0.95).

## Methods

### Study design

In this study, we developed a machine learning-based tool that predicts an individual’s 3-year risk of AS, herein ASrisk, using only blood sample data and vital measurements. We trained, tested and externally validated this tool using 55 clinical features from the EHR of two large-scale longitudinal cohorts. We addressed the TRIPOD + AI checklist for the reporting of prediction model studies.^19^

### Study participants

We trained, tested and evaluated the predictive capacity of ASrisk using EHR from the Mount Sinai Data Warehouse (MSDW), a hospital-based, multiethnic cohort comprised of over 11 million patient records and 87 million patient encounters of individuals attending the Mount Sinai Health System in New York, NY. To externally validate ASrisk, we used EHR from the UK Biobank, a population-based cohort of 502 505 predominantly European individuals between the ages of 40 and 69 years old.^20^ Family history, lifestyle factors, health status, physician-diagnosed medical conditions, as well as laboratory and physical measurements were obtained at recruitment between 2006 and 2010 in 22 assessment centres established across the United Kingdom. Study protocols were approved by the Institutional Review Board at the Icahn School of Medicine at Mount Sinai (New York City, NY, USA; GCO #07-0529; STUDY-11-01139). Use of data from the UK Biobank was approved with the UK Biobank Resource under application number 16218.

### Clinical outcomes

In the MSDW, we defined AS cases using International Classification of Diseases (ICD)-10 code I35.0. We defined controls likely to be undiagnosed cases (hereinafter, excluded controls) using ICD-10 codes I08.0, I71.0, I71.21, I71.9, I77.81, I35.1, Q23.1, Q23.0, Q24.4, Q25.3, and Z95.2, and prescriptions of anticoagulants, antiplatelets, beta-blockers, diuretics, inhibitors of *SGLT2*, anti-hypertensives, and lipid-lowering medication. We defined AS controls as all individuals without any of the above-listed ICD-10 codes. We defined cases of CAD using ICD-10 codes I21-I25, hypertensive heart disease (HHD) using ICD-10 codes I10, I11, stroke using ICD-10 code I63, chronic kidney disease (CKD) using ICD-10 code N18, dementia using ICD-10 codes F00-F03, G30, and AVR using ICD-10 code Z95.2. We used the following thresholds to define clinical severe AS findings in echocardiographic data: <1.0 cm^2^ for aortic valve area, <0.25 for Doppler velocity index, >40 mmHg for mean gradient, and >4.0 m/s for peak velocity. In the UK Biobank, we defined AS cases using ICD-10 code I35.0. We did not define excluded controls in the UK Biobank because we only evaluated the performance of the AS prediction tool in an open cohort setting; hence, all individuals without ICD-10 code I35.0 were considered controls. We defined cases of AVR using ICD-10 code Z95.2 and Operating Procedure Codes Supplement (OPCS)-4 codes K26.1, K26.2, K26.3, and K26.4.

### Development of the AS score

**Figure 1:**
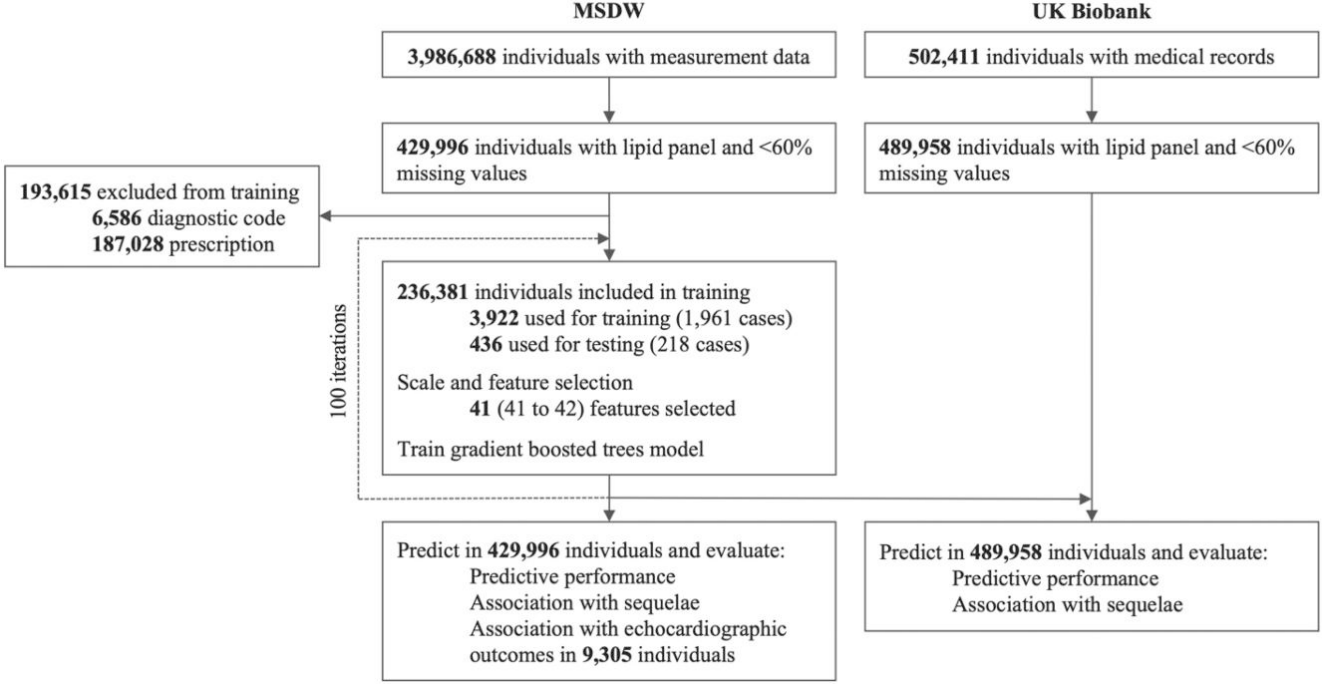
Study design. We trained and tested machine learning models for 3-year aortic stenosis risk prediction in the Mount Sinai Data Warehouse (MSDW). We then used these models to calculate ASrisk in all individuals of MSDW and the UK Biobank.

We used blood sample data and vital sign measurements from the EHR of individuals registered in the MSDW to train and test machine learning models for 3-year risk prediction of AS. To build a 3-year predictive model, we masked the EHR of AS cases 3 years prior to diagnosis. As a result, all individuals are free from AS at baseline and at the time of model training, testing, and evaluation. We removed AS cases with <3 years of data prior to diagnosis and removed excluded controls. This ensures that all individuals used for training and testing were free from AS at baseline. We adapted this framework to generate portable risk prediction tools from previous studies of cardiovascular risk prediction and characterization using EHR and ML.^10,11^ We removed clinical features and individuals with >60% missing values. To minimize sampling bias, we repeated the workflow 100 times randomly sampling individuals used for training and testing in each iteration. The ML workflow is described below for a single iteration (*Figure 1*). We randomly selected 90% of cases and an equal number of controls to create the train set. Then, we used the remaining 10% of cases and an equal number of controls to create the test set. To collapse longitudinal data, we used the median of multiple datapoints for a given feature in 1-year margin prior to the last entry date in all individuals. Given that the MSDW is a hospital-based cohort with greater prevalence of disease, we removed controls with signs of possible underdiagnosis from training to minimize misclassification (see ‘clinical outcomes’ for definitions). To make the prediction task clinically interpretable, we reduced the complexity of the model by performing feature selection^21^ on the train set. Age and gender were used as covariates and therefore not considered during feature selection. Furthermore, we only considered features present both in the MSDW and the UK Biobank to facilitate external validation. Non-selected features were removed from the test set accordingly. We used the resulting datasets to train and test a gradient boosted trees model^22^ using the caret package^23^ in R. We optimized hyperparameters using an internal 10-fold cross-validation within the train set. We repeated this workflow 100 times, resulting in 100 independent machine learning models for 3-year AS risk prediction. Finally, we used these 100 models trained in MSDW to compute ASrisk for all individuals in the MSDW, including ‘excluded’ controls, and in the UK Biobank. The prediction for each individual results from the average of 100 independent predictions. All individuals in the MSDW and UK Biobank are free from AS at the time of model training, testing and evaluation.

### Evaluation of the AS score

First, we evaluated the capacity of ASrisk to predict AS diagnosis 3 years in advance in the general population. For that, we calibrated the ASrisk score to the risk distribution of all individuals in MSDW and the UK Biobank using the rms package version 6.8-1.^24^ We then used this calibrated ASrisk score to evaluate its predictive performance on balanced sets of AS cases and controls in both cohorts. For this, we used an arbitrary threshold of ASrisk > 0.5 to define positive predictions given that the baseline hazard of AS risk is 50% in the balanced sets. This threshold and the resulting performance metrics do not represent the use of ASrisk in clinical practice. Furthermore, to test whether ASrisk prediction capture AS pathophysiology specifically, we examined which features drive >50% of the tool’s prediction, and tested ASrisk for association with diagnoses that could potentially show a similar risk factor profile in EHR (namely CAD, HHD, CKD, dementia, and stroke). We adjusted the latter model for age and sex and removed AS cases from the analysis to limit confounding of the outcome label.

Second, we evaluated the clinical information entailed by higher deciles of the AS score in the general population. For this, we tested deciles of ASrisk for association with AS sequelae (defined as AVR) in MSDW and the UK Biobank.

Third, we tested whether higher values of the ASrisk score track with echocardiographic findings that inform AS diagnosis. For that, we collected aortic valve area, Doppler velocity index, mean gradient, peak velocity, and AS severity assessment in MSDW. We defined positive findings as aortic valve area < 1.0 cm^2^, Doppler velocity index < 0.25, mean gradient > 40 mmHg, and peak velocity > 4.0 ms^−1^. We only considered echocardiographic information at the time of model training for every participant. Hence, AS cases were not diagnosed with AS at the time of echocardiographic screening. Furthermore, individuals with echocardiographic screening generally have signs of cardiovascular diseases, which can lead to prescription biases. To leverage this, we incorporated individuals with encounter for screening for cardiovascular disorders (defined by ICD-10 code Z13.6) and no echocardiographic information. We then tested association of ASrisk with echocardiographic findings as defined above.

Fourth, we evaluated the potential use of ASrisk to identify asymptomatic high-risk individuals in the general population. For that, we evaluated enrichment of AS diagnosis after 3 years of risk assessment, and average reduction in aortic valve area between risk assessment and AS diagnosis, at increasing thresholds of ASrisk (from 0.70 to 0.95).

### Statistical analyses

We performed all statistical analyses in R.^25^ We evaluated discrimination capacity of ASrisk in MSDW and the UK Biobank using the receiver operating characteristic (ROC) curve encompassed in pROC version 1.14.0.^26^ Furthermore, we assessed the predictive performance as described by sensitivity, specificity, negative predictive value (NPV), positive predictive value (PPV), and overall accuracy using the R caret package version 6.0.84.^23^ And finally, we calibrated ASrisk using the rms package version 6.8-1.^24^

## Results

### Description of the study population

The study population included 919 954 individuals from two large scale longitudinal cohorts used to develop and evaluate ASrisk. We used EHR from 236 381 individuals in MSDW (2179 AS cases and 234 202 controls without exclusion criteria) to train and test the machine learning models. We then used EHR from 429 996 individuals in MSDW to compute ASrisk [median age 56 years (38–71), 241 070 females (56%), and 2179 AS cases (0.5%)] (*Table 1*). In line with the low prevalence of AS, the majority of individuals in MSDW are predicted to have low long-term disease risk [median ASrisk 0.015 (0.0015–0.15)] (see Supplementary material online, *Figure S1*); while higher deciles of the score show larger proportions of AS cases (see Supplementary material online, *Figure S2*). Furthermore, median ASrisk was greater in AS cases compared with controls by 0.45 (median ASrisk 0.46 in cases vs. 0.014 in controls). To evaluate the tool’s performance in an external population-based cohort, we used EHR from 489 958 individuals in the UK Biobank [median age 57 years (50–63), 218 749 females (57%), and 5066 AS cases (1.0%)] (*Table 1*). The distribution of ASrisk and proportion of AS cases at increasing deciles of the score is similar in the UK Biobank compared with MSDW (see Supplementary material online, *Figures S1* and *S2*).

**Table 1:** Baseline characteristics of the mount Sinai data warehouse and UK biobank cohorts

Characteristic	MSDW	UK Biobank
Cases, *n* (%)	2179 (0.5)	5066 (1.0)
Age, median (IQR)	56 (38–71)	57 (50–63)
Female, *n* (%)	241 070 (56.0)	218 749 (57.3)
Systolic blood pressure, median (IQR)	123 (114–132)	137 (125–150)
LDL cholesterol, median (IQR)	99 (81–122)	104 (89–119)
HDL cholesterol, median (IQR)	53 (44–65)	55 (47–66)
Triglycerides, median (IQR)	103 (75–145)	129 (92–185)
Hypertension diagnosis, *n* (%)	133 113 (30.9)	53 659 (23.5)
Diabetes diagnosis, *n* (%)	35 462 (8.3)	14 453 (6.0)
Heart failure diagnosis, *n* (%)	18 319 (4.3)	16 491 (3.4)
Stroke diagnosis, *n* (%)	8512 (2.0)	8665 (1.8)
Arrythmia diagnosis, *n* (%)	3406 (0.8)	2681 (0.5)
Aortic valve replacement, *n* (%)	2205 (0.5)	3777 (0.8)
Clinical risk score, median (IQR)	1.5 (0.2–14.6)	6.2 (2.4–15.4)

We measured baseline population characteristics in 429 996 individuals from the Mount Sinai Data Warehouse and 489 958 individuals from the UK Biobank.

MSDW, Mount Sinai Data Warehouse; LDL, low-density lipoprotein; HDL, high-density lipoprotein.

### Predictive performance of the AS score

In the internal training cohort, the machine learning models predicted 3-year AS risk with an AUROC of 0.90 [standard error (SE) = 0.0043] in the training dataset and 0.90 (SE = 0.014) in the testing dataset (see Supplementary material online, *Table S1* and Supplementary material online, *Figure S3*). When evaluating the performance of ASrisk in an open cohort study where all MSDW individuals are included to replicate the expected incidence of AS in hospitals, the tool predicts AS diagnosis with an AUROC of 0.87 (SE = 0.0048) (*Table 2* and Supplementary material online, *Figure S4*). Importantly, 84% of individuals with ASrisk > 0.5 will be diagnosed with AS after 3 years (PPV = 0.84). In an open cohort study where all individuals in the UK Biobank are included to replicate the expected incidence of AS in the general population, ASrisk predicts AS diagnosis with similar precision (AUROC = 0.72 and PPV = 0.81) (*Table 2* and Supplementary material online, *Figure S4*). ASrisk shows improved discrimination of AS cases and controls compared with a recently published AS risk prediction model by 10% in the derivation cohort (discrimination of 0.87 for ASrisk vs. 0.77 for the statistical model) and 8% in the external validation cohort (discrimination of 0.72 for ASrisk vs. 0.64 for the statistical model).^27^ Feature importance analyses and Shapley additive explanation (SHAP) values revealed that biomarkers of AS progression or severity, such as haemoglobin A1c,^28^ systolic blood pressure,^29^ and albumin,^30^ heavily contribute to risk prediction (see Supplementary material online, *Table S2* and Supplementary material online, *Figure S5*). Furthermore, negative or weak association with conditions that can show similar risk factor profiles in the EHR demonstrate that ASrisk captures AS pathophysiology specifically (see Supplementary material online, *Table S3*).

**Table 2:** Performance metrics mount Sinai data warehouse and the UK biobank

	AUROC	Accuracy	Sensitivity	Specificity	PPV	NPV	PLR	NLR
MSDW	0.87 (0.0048)	0.68 (0.0030)	0.45 (0.00)	0.92 (0.0060)	0.84 (0.0094)	0.63 (0.0015)	5.63 (0.42)	0.60 (0.0039)
UK Biobank	0.72 (0.0036)	0.56 (0.0013)	0.16 (0.00)	0.96 (0.0026)	0.81 (0.011)	0.53 (0.0068)	4.00 (0.26)	0.88 (0.0024)

We averaged (standard deviation) performance metrics across 100 machine learning models using electronic health records from 429 996 individuals in Mount Sinai Data Warehouse and 489 958 individuals in the UK Biobank. We used a threshold of ASrisk > 0.5 to define positive predictions.

AUROC, area under the receiver operator characteristic curve; PPV, positive predictive value; NPV, negative predictive value; PLR, positive likelihood ratio; NLR, negative likelihood ratio; MSDW, Mount Sinai Data Warehouse.

### Associations with aortic stenosis sequelae

We then evaluated signs of AS pathophysiology represented by higher deciles of ASrisk. For this, we tested deciles of ASrisk for association with presence of AS sequelae. In MSDW, the odds of AVR procedures increased by 63% with each increase in decile of the score (odds ratio [OR] 1.63 (95% CI 1.60–1.67), *P* < 0.0001) (see Supplementary material online, *Table S4*). Similarly, individuals in higher deciles of ASrisk show increased prevalence and odds of AVR in the UK Biobank [OR 1.27 (95% CI 1.25–1.28), *P* < 0.0001] (see Supplementary material online, *Table S4*). This shows that higher ASrisk entails signatures of AS pathophysiology.

### Associations with echocardiographic findings

We then evaluated whether ASrisk tracks with echocardiographic findings used for AS diagnosis. For this, we tested ASrisk for association with echocardiographic outcomes in MSDW. Association analyses demonstrated that ASrisk tracks with signs of severe aortic valve stenosis, such as a Doppler velocity index lower than 0.25 [OR 1.88 (95% CI 1.71–2.06), *P* < 0.0001] (*Table 3*). Similarly, higher ASrisk is associated with clinical findings of AS severity, such as aortic valve area by velocity time integral <1.0 cm^2^ [OR 2.50 (95% CI 2.36–2.64), *P* < 0.0001], mean gradient higher than 40 mmHg [OR 2.61 (95% CI 1.89–2.71), *P* < 0.0001], and peak velocity higher than 4.0 m s^−1^ [OR 1.98 (95% CI 1.72–2.29), *P* < 0.0001] (*Table 3*). Finally, a SD increase in ASrisk increases the odds of mild [OR 2.65 (95% CI 2.50–2.81), *P* < 0.0001], moderate [OR 2.04 (95% CI 1.89–2.21), *P* < 0.0001], and severe [OR 1.98 (95% CI 1.83–2.14), *P* < 0.0001] aortic valve stenosis as assessed by a board-certified echocardiographer (*Table 3*). Overall, associations with echocardiographic data suggest that undiagnosed individuals with higher ASrisk are more likely to present with positive findings in diagnostic testing for AS.

**Table 3:** Associations of echocardiographic findings with ASrisk in mount Sinai data warehouse

Echocardiographic entry	OR	Lower 95% CI	Upper 95% CI	*P*
Doppler velocity index	1.88	1.71	2.06	5.60e-40
Aortic valve area by VTI	2.50	2.36	2.64	2.75e-223
Mean gradient	2.61	1.89	2.71	6.25e-19
Peak velocity	1.98	1.72	2.29	1.28e-20
Mild AVD	2.65	2.50	2.81	3.31e-244
Moderate AVD	2.04	1.89	2.21	8.40e-71
Severe AVD	1.98	1.83	2.14	2.42e-67

We tested ASrisk for association with presence of diagnostic finding in echocardiographic data.

OR, odds ratio; CI, confidence interval; VTI, velocity time integral; AVD, aortic valve disease.

### Clinical use

**Figure 2:**
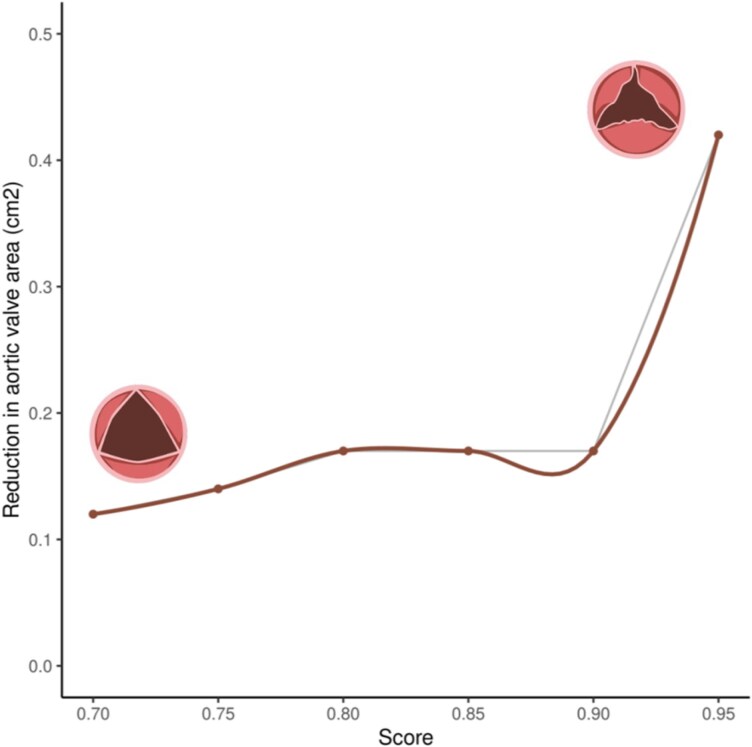
Reduction in aortic valve area (cm^2^) between risk assessment and aortic stenosis diagnosis in high-risk individuals. We calculated the average reduction in aortic valve area between risk assessment and aortic stenosis diagnosis at least 3 years later in aortic stenosis cases from the Mount Sinai Data Warehouse with echocardiographic information. The average reduction in aortic valve area corresponds to individuals above the threshold of ASrisk defined in the *x*-axis.

Finally, we evaluated the clinical use of ASrisk by assessing enrichment in 3-year AS diagnosis at increasing thresholds of ASrisk. This demonstrated that individuals with ASrisk > 0.95 have 10.93 (95% CI 6.97–16.27, *P* < 0.0001) and 11.35 (95% CI 5.29–21.45, *P* < 0.0001) higher odds of AS diagnosis after 3 years compared with individuals below that threshold in MSDW and the UK Biobank, respectively (*Table 4*). Additionally, individuals with ASrisk > 0.95 suffer an average reduction of 0.42 cm^2^ in their aortic valve area between the date of risk assessment and the date of AS diagnosis, compared with 0.12 cm^2^ in individuals with ASrisk > 0.70 (*Figure 2*). Overall, these results suggest ASrisk can be used to highlight asymptomatic individuals at high risk of AS.

**Table 4:** Enrichment of aortic stenosis cases at increasing thresholds of ASrisk in mount Sinai data warehouse and the UK biobank

	Mount Sinai Data Warehouse				UK Biobank			
Threshold	OR	Lower 95% CI	Upper 95% CI	*P*	OR	Lower 95% CI	Upper 95% CI	*P*
0.70	8.00	7.22	8.84	2.20e-316	6.56	5.81	7.38	1.52e-208
0.75	8.07	7.22	9.00	7.04e-300	7.09	6.13	8.15	1.64e-160
0.80	8.41	7.40	9.52	4.35e-241	7.19	5.97	8.59	1.47e-100
0.85	8.76	7.49	10.18	4.63e-169	8.78	6.91	11.00	2.78e-75
0.90	9.73	7.81	11.98	7.17e-97	11.77	8.38	16.08	7.18e-50
0.95	10.93	6.97	16.27	1.11e-28	11.35	5.29	21.45	5.73e-12

We tested enrichment of aortic stenosis cases at least 3 years after risk assessment in high-risk individuals, defined as above a certain threshold of ASrisk (from 0.70 to 0.95) in Mount Sinai Data Warehouse and the UK Biobank.

OR, odds ratio; CI, confidence interval.

## Discussion

In this study, we evaluated the performance of a machine learning-based tool to predict long-term risk of AS in two large-scale longitudinal cohorts. This revealed four significant findings. First, machine learning models trained on serum biomarkers and vital sign measurements can capture the risk and severity of AS 3 years ahead of diagnosis. Second, the AS risk score tracks diagnostic findings in high-risk individuals and accurately quantifies the severity of AS on echocardiograms. Third, sequelae of AS, defined by AVR, is more prevalent at higher deciles of the AS risk score. Last, high-risk individuals (ASrisk > 0.95) have 11-fold higher chances of AS diagnosis 3 years after risk assessment and an average 3-year reduction in aortic valve area of 0.42 cm^2^.

Machine learning has been used to develop prediction models for the diagnosis of AS.^12,14^ However, these studies rely on electrocardiograms or echocardiograms which remain largely limited to emergency rooms, inpatient hospitalizations, or cardiology clinic practices, and are generally prescribed to individuals with suspicion of cardiovascular disease. To our knowledge, this study is the first study using serum biomarkers and vital sign measurements to predict risk of AS in the general population. The use of routine clinical data can address needs in clinical practice that are unfeasible due to unavailability of complex diagnostic tests. First, it allows for AS risk estimations in the general population, with the potential to identify asymptomatic individuals who could go on to develop significant AS. Second, it can be more broadly applied to a wide range of clinical practices, including primary care settings, which are often the first interaction patients have with physicians. Third, the broad use of serum biomarkers and vital sign measurements makes it portable to external health systems or resource poor settings. Hence, ASrisk addresses a gap in clinical practice by enabling scalable and portable estimations of AS risk in the general population.

ASrisk shows potential to identify future cases of AS at a minimal false positive rate. As shown in our study, higher deciles of ASrisk track severity of AS and reduction in aortic valve area, as well as progression of AS otherwise captured with echocardiographic examinations. Thus, higher deciles of ASrisk can help identify patients who could warrant an echocardiogram regardless of physical exam findings in the general population. Similarly, an increase in ASrisk is associated with AVR in the general population. This suggests ASrisk may help inform actionable preventive strategies in a population of individuals that are missed by the current standard of care. Overall, ASrisk can be deployed horizontally throughout clinical practices to identify patients at risk for severe AS regardless of clinical findings. This score can potentially tailor echocardiographic examination to a population of individuals at high risk of AS. Of note, categorical performance metrices (e.g. PPV or NPV) reported in this study do not reflect the use of ASrisk in clinical practice. Prospective evaluation of the tool is needed to determine the performance of ASrisk in clinics. To facilitate this, we have deposited the codes needed to develop and evaluate ASrisk at https://github.com/rondolab/ASrisk. The code is built to work in any digitalized healthcare systems using the OMOP Common Data Model. Additionally, ASrisk needs to be internally evaluated to adjust its risk threshold and calibration to each population’s baseline hazard.

Finally, our analyses indicate that over 50% of the model’s prediction is determined by a combination of clinical and biochemical features, namely haemoglobin A1c, systolic blood pressure, albumin, basophil percentage, red blood cell count, glucose and platelet count. Their predictive signal for AS is likely a combination of their individual association with 3-year AS diagnosis, but also their interaction throughout the patient’s clinical history. For instance, elevated HbA1c and glucose signify chronic and acute hyperglycaemia, which can drive endothelial dysfunction and inflammatory processes around the aortic valve,^31^ promoting lipid infiltration and oxidative stress that can initiate early valvular calcification. Reduced red blood cell and platelet counts can be indicators of increased AS severity due to shearing of platelets causing bleeding through intestinal arteriovenous malformation.^32–34^ High systolic blood pressure exacerbates mechanical stress, while low albumin often reflects poor nutritional status and frailty, commonly seen in an elderly population with the highest prevalence of severe degenerative AS.^35,36^ Finally, basophils, though present in small numbers, release mediators that perpetuate local inflammation leading to aortic valve calcification.^37,38^ The role of these features in ASrisk demonstrate that a combination of clinical and biochemical markers can capture AS pathophysiology and can therefore be used for early identification of AS in combination with current prognostic variables derived from electrocardiogram or echocardiogram data.

## Limitations

Our study has limitations. First, AS case status was obtained using diagnostic codes, which might lead to misclassification. Although we used ICD-10 codes specifically for non-rheumatic AS and excluded ICD-10 codes that could confound our model such as bicuspid aortic valve and rheumatic AS, the potential for misclassification bias is present in our model. Second, we had a limited number of patients with echocardiographic data compared with the total number of clinical encounters used to train our model. Regardless, our model was strongly associated with all echocardiographic criteria for severity of AS in the validation set. One of the limitations is that we were unable to validate our model in the UK Biobank since no echocardiographic data is available as of today.

## Conclusions

In conclusion, we leveraged machine learning and clinical EHR data to derive and validate a model to predict AS in the general population 3 years before diagnosis. The model was able to capture severity of AS on a continuous spectrum and correlates well with echocardiographic data and clinical outcomes. Further research is required to assess the performance of this in-silico marker in prospective studies.

## Supplementary Material

ztag035_Supplementary_Data

## Data Availability

The codes to build 3-year AS risk prediction models using routine clinical data from digitalized healthcare systems and compute ASrisk across the entire population can be accessed at https://github.com/rondolab/ASrisk. These codes will be made publicly available upon publication. The dataset from UK Biobank analysed in the study is available via application number 16218 to the Access Management System at https://ams.ukbiobank.ac.uk/ams/. Further information regarding the Mount Sinai Data Warehouse is available at https://labs.icahn.mssm.edu/msdw/.
