# Supplementary material for: Three-year risk prediction of aortic stenosis using routine medical records: derivation and validation in 919 954 individuals from two cohorts
Source: Eur Heart J Digit Health. 2026 Mar 2;7(3):ztag035. doi: 10.1093/ehjdh/ztag035 (PMC12975180; doi:10.1093/ehjdh/ztag035)
Supplement: ztag035_Supplementary_Data [file ztag035_supplementary_data.docx]

**Title:** Three-year risk prediction of aortic stenosis using routine medical records: Derivation and validation in 919,954 individuals from two cohorts.

**Authors:** Ben O. Petrazzini^1-4*^, BS, Waqas A. Malick^5*^, MD, Stamatios Lerakis^6,7^, MD, Lori B. Croft^6^, MD, Ghislain Rocheleau^1-3^, PHD, Robert S. Rosenson^5#^, MD, Ron Do^1-3#^, PHD.

**Affiliations:**

1. The Charles Bronfman Institute for Personalized Medicine, Icahn School of Medicine at Mount Sinai, New York, NY, USA.
2. Department of Genetics and Genomic Sciences, Icahn School of Medicine at Mount Sinai, New York, NY, USA.
3. The Windreich Department of Artificial Intelligence and Human Health, Icahn School of Medicine at Mount Sinai, New York, NY, USA.
4. Deep Medicine, Nuffield Department of Women’s and Reproductive Health, University of Oxford, Oxford, UK.
5. Metabolism and Lipids Program, Mount Sinai Fuster Heart Hospital, Icahn School of Medicine at Mount Sinai, New York, NY, USA.
6. Mount Sinai Fuster Heart Hospital, Icahn School of Medicine at Mount Sinai, New York, NY, USA.
7. Department of Cardiology, Mount Sinai Morningside Hospital, Icahn School of Medicine at Mount Sinai, New York, NY, USA.

* Contributed equally

# Jointly supervised the study

**Supplementary Tables:**

**Supplemental Table 1.** Performance metrics for training and testing in Mount Sinai Data Warehouse.

**Supplemental Table 2.** Contribution of clinical features to risk prediction in the machine learning models.

**Supplementary Table 3.** Association of ASrisk with diagnoses.

**Supplementary Table 4.** Association of aortic stenosis sequelae with the ASrisk.

**Supplementary Figures**

**Supplementary Figure 1.** Distribution of ASrisk in Mount Sinai Data Warehouse and the UK Biobank

**Supplementary Figure 2.** Percentage of aortic stenosis cases in deciles of ASrisk in Mount Sinai Data Warehouse and the UK Biobank

**Supplementary Figure 3.** Area under the receiver operator characteristic curve for training and testing in Mount Sinai Data Warehouse.

**Supplementary Figure 4.** Area under the receiver operator characteristic curve in Mount Sinai Data Warehouse and the UK Biobank.

**Supplementary Figure 5.** Shapley additive explanations (SHAP) values for clinical features used to train the machine learning models.

**Supplementary Table 1.** Performance metrics for training and testing in Mount Sinai Data Warehouse.

|  | AUROC | Accuracy | Sensitivity | Specificity | PPV | NPV | PLR | NLR |
| --- | --- | --- | --- | --- | --- | --- | --- | --- |
| Train | 0.90 (0.0043) | 0.83 (0.0042) | 0.85 (0.0060) | 0.80 (0.0068) | 0.81 (0.0052) | 0.84 (0.0052) | 4.25 (0.15) | 0.19 (0.0077) |
| Test | 0.90 (0.014) | 0.82 (0.016) | 0.84 (0.019) | 0.79 (0.027) | 0.80 (0.021) | 0.83 (0.017) | 4.00 (0.52) | 0.20 (0.025) |

We tested the performance of the machine learning models in predicting cases of AS in the balanced training and testing cohorts designed to limit leakage of AS cases into the control label. Performance metrics are the average (standard deviation) for 100 models trained using electronic health records from 236,381 individuals in the Mount Sinai Data Warehouse. We used a threshold of ASrisk>0·5 to define positive predictions. Train refers to performance metrics in the internal 10-fold cross validation performed during training. Test refers to performance metrics in the hold-out test set. AUROC, area under the receiver operator characteristics curve; PPV, positive predictive value; NPV, negative predictive value; PLR, positive likelihood ratio; NLR, negative likelihood ratio.

**Supplementary Table 2.** Contribution of clinical features considered to risk prediction in the machine learning models.

| Feature | Importance (%) |
| --- | --- |
| Hemoglobin A1c | 17.78 |
| Systolic BP | 13.97 |
| Albumin | 7.94 |
| Basophil % | 5.43 |
| RBC | 3.93 |
| Glucose | 3.69 |
| Platelet | 3.63 |
| Diastolic BP | 3.41 |
| Pulse | 2.93 |
| Creatinine | 2.76 |
| Lymphocyte % | 2.65 |
| Weight | 2.07 |
| BMI | 2.05 |
| Monocyte # | 2.03 |
| Age | 1.95 |
| ALK phosphatase | 1.82 |
| Red distr. width | 1.78 |
| Height | 1.35 |
| Monocyte % | 1.34 |
| Mean corp. HGB | 1.34 |
| Mean corp. vol. | 1.33 |
| LDL cholesterol | 1.30 |
| ALT SGPT | 1.14 |
| AST SGOT | 1.09 |
| HDL cholesterol | 1.06 |
| Mean platelet vol. | 1.05 |
| Eosinophil (%) | 0.99 |
| Calcium | 0.95 |
| Triglycerides | 0.92 |
| Neutrophil (#) | 0.84 |
| Mean corp. hgb. con. | 0.78 |
| Total protein | 0.77 |
| WBC count | 0.67 |
| Neutrophil (%) | 0.66 |
| Bilirubin total | 0.64 |
| Hematocrit | 0.58 |
| Hemoglobin | 0.52 |
| Eosinophil (#) | 0.39 |
| Lymphocyte (#) | 0.35 |
| Basophil (#) | 0.05 |
| Gender | 0.03 |

We average the feature importance across 100 models trained in the Mount Sinai Data Warehouse. Show are all clinical features considered by the machine learning models after feature selection. BP, blood pressure; RBC, red blood cell count; BMI, body mass index; ALK, alkaline, HGB, haemoglobin.

**Supplementary Table 3.** Association of ASrisk with diagnoses.

| Outcome | OR | Lower 95% CI | Upper 95% CI | *P* | Adjustment |
| --- | --- | --- | --- | --- | --- |
| AS | 25.12 | 21.39 | 29.41 | <2.20e-316 | Age and gender |
| AS | 27.91 | 22.66 | 34.32 | 4.02e-221 | Age, gender and diagnoses of CAD, HHD, CKD, dementia and stroke |
| CAD | 0.55 | 0.37 | 0.83 | 0.0031 | Age, gender and AS status |
| HHD | 0.42 | 0.29 | 0.60 | 4.90e-6 | Age, gender and AS status |
| CKD | 2.62 | 1.49 | 4.60 | 0.0084 | Age, gender and AS status |
| Dementia | 1.14 | 0.51 | 2.55 | 0.75 | Age, gender and AS status |
| Storke | 1.21 | 0.59 | 2.49 | 0.59 | Age, gender and AS status |

We tested association of ASrisk with aortic stenosis (AS), coronary artery disease (CAD), hypertensive heart disease (HHD), chronic kidney disease (CKD), dementia and stroke in the Mount Sinai Data Wearhouse (MSDW). OR, odds ratio; CI, confidence interval.

**Supplementary Table 4.** Association of aortic stenosis sequelae with the ASrisk.

|  | OR | Lower 95% CI | Upper 95% CI | *P* |
| --- | --- | --- | --- | --- |
| MSDW | 1.63 | 1.60 | 1.67 | <2.20e-316 |
| UK Biobank | 1.27 | 1.25 | 1.28 | 1.69e-290 |

We tested association of aortic valve replacement with deciles of ASrisk in the Mount Sinai Data Wearhouse (MSDW) and UK Biobank. OR, odds ratio; CI, confidence interval.

**
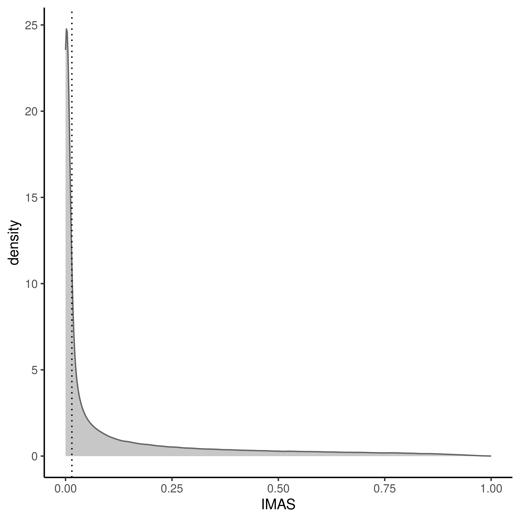

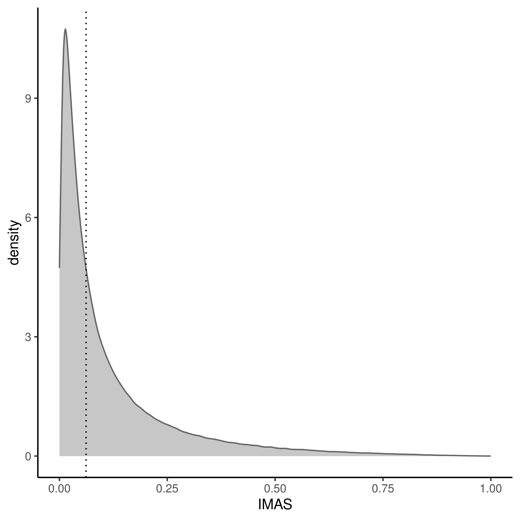
Supplementary Figure 1.** Distribution of ASrisk in Mount Sinai Data Warehouse and the UK Biobank

A

B

We plotted the distribution of ASrisk in 429,996 individuals from the Mount Sinai Data Warehouse (A) and 489,958 individuals from the UK Biobank (B).

**
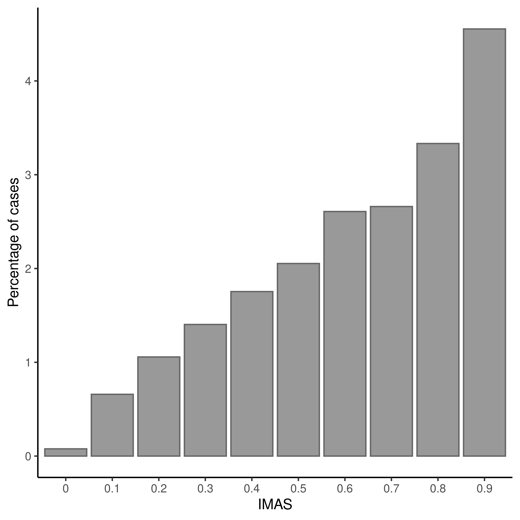

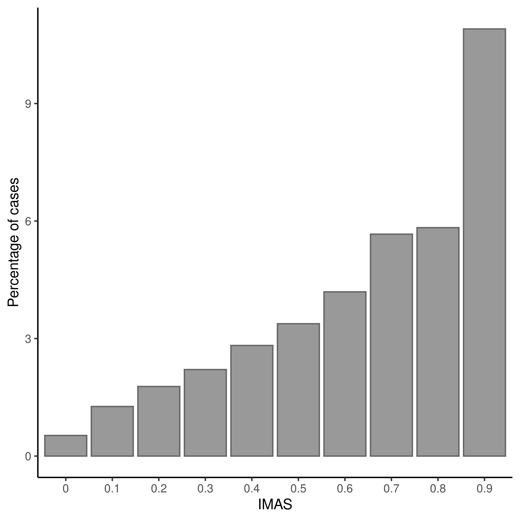
Supplementary Figure 2.** Percentage of aortic stenosis cases in deciles of ASrisk in Mount Sinai Data Warehouse and the UK Biobank

A

B

We calculated the percentage of cases of aortic stenosis in increasing deciles of ASrisk in 429,996 individuals from the Mount Sinai Data Warehouse (A) and 489,958 individuals from the UK Biobank (B).

**
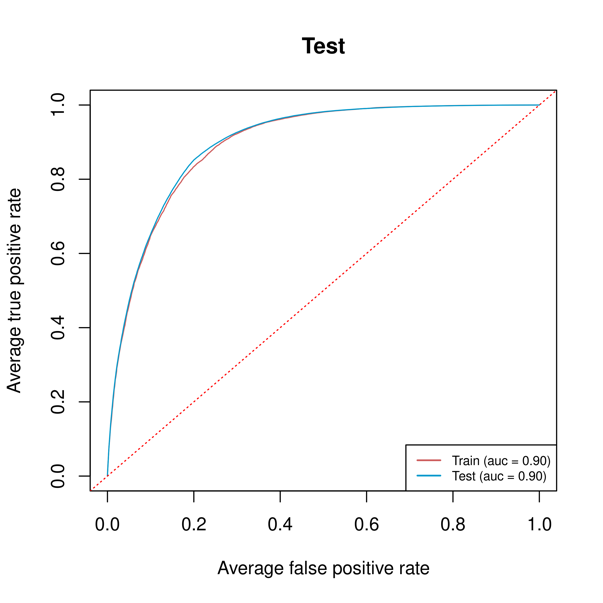
Supplementary Figure 3.** Area under the receiver operator characteristic curve for training and testing in Mount Sinai Data Warehouse.

We plotted the averaged area under the receiver operator characteristic curve across 100 models trained using electronic health records from 236,381 individuals in the Mount Sinai Data Warehouse. Train refers to performance metrics in the internal 10-fold cross validation performed during training. Test refers to performance metrics in the hold-out test set. AUC, area under the curve.

**Supplementary Figure 4.** Area under the receiver operator characteristic curve in Mount Sinai Data Warehouse and the UK Biobank.

**
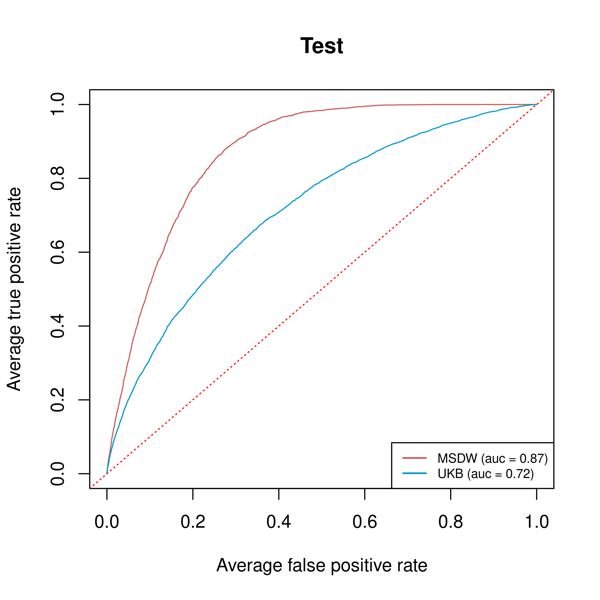
**

We calculated the area under the receiver operator characteristic curve for 100 models using 429,996 individuals from Mount Sinai Data Warehouse and 489,958 individuals from the UK Biobank and plotted the average estimation across 100 models. MSDW, Mount Sinai Data Warehouse; UKB, UK Biobank.


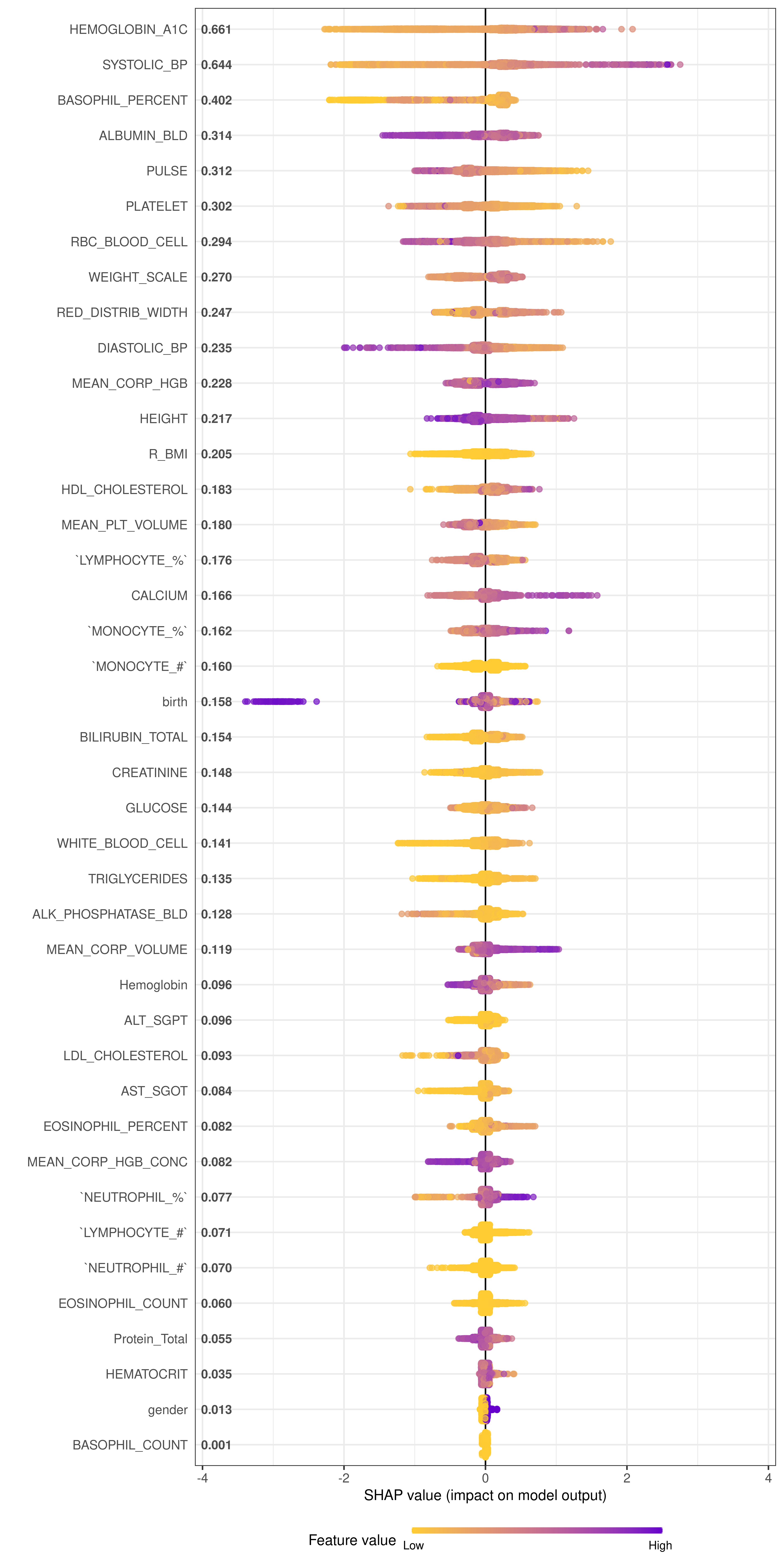
**Supplementary Figure 5.** Shapley additive explanations (SHAP) values for clinical features used to train the machine learning models.

We calculated shapley additive explanation (SHAP) values for 100 models trained in the Mount Sinai Data Warehouse. The birth feature indicates age of participant. BP, blood pressure; RBC, red blood cell count; BMI, body mass index; ALK, alkaline, HGB, haemoglobin.
